# Protective effect and mechanism insight of purified Antarctic krill phospholipids against mice ulcerative colitis combined with bioinformatics

**DOI:** 10.1007/s13659-023-00375-2

**Published:** 2023-04-05

**Authors:** Rong Huang, Jiaxu Yao, Li Zhou, Xiang Li, Jinrui Zhu, Yueqi Hu, Jikai Liu

**Affiliations:** National Demonstration Center for Experimental Ethnopharmacology Education, School of Pharmaceutical Sciences, South-Central MinZu University, Wuhan, 430074 People’s Republic of China

**Keywords:** Ulcerative colitis, Krill phospholipids, Bioinformatics analysis, Tight junction protein, Intestinal microbiota

## Abstract

Antarctic krill oil is functional oil and has a complex phospholipids composition that poses difficulties in elucidating its effect mechanism on ulcerative colitis (UC). The mechanism of UC action was studied by bioinformatics, and the therapeutic effect of Antarctic krill phospholipids (APL) on dextran sulfate sodium (DSS)-induced colitis mice was verified. GO functional enrichment analysis uncovered an enrichment of these genes in the regulation of cell–cell adhesion, membrane region, signaling receptor activator activity, and cytokine activity. Meanwhile, the KEGG results revealed the genes were enriched in the TNF signaling pathway, pathogenic *Escherichia coli* infection, inflammatory bowel disease and tight junction. Animal experiments showed that APL treatment alleviated the UC symptoms and reduced inflammatory damage. Meanwhile, the expressions of the tight junction (TJ) proteins, ZO-1 and occludin, were restored, and the levels of IL-6 and TNF-α were reduced. Moreover, *Firmicutes*/*Bacteroidetes* ratio in the intestinal microbiota was regulated, and the contents of short-chain fatty acids metabolites were raised. These findings would provide an insight for the beneficial effects of APL and dietary therapy strategies for UC.

**Supplementary Information:**

The online version contains supplementary material available at 10.1007/s13659-023-00375-2.

## Introduction

As one of the modern refractory diseases, ulcerative colitis (UC) is chronic inflammation in the human gastrointestinal tract [1], which focus is concentrated in the colonic mucosa [2]. The annual incidence of UC is around 10–12 per 100,000 people, with the age of onset peaking at 15–25 and 55–65 years old [3]. Weight loss, abdominal pain, and diarrhea are the main clinical signs of UC. The relapsing–remitting process of the disease causes a dramatic decrease in the patient's life quality [4]. In clinical practice, therapeutic drugs are chosen based on the stage of the disease. Mild-moderate UC patients prefer aminosalicylic acid and glucocorticoids, while immunosuppressants and biologics are provided for moderate-severe patients [5, 6]. However, due to the long disease course and recurrent attacks of UC, deficiencies in drug treatment still exist. On account of the therapeutic efficacy of oral aminosalicylic acid heavily depends on patient compliance, only 40–60% of patients ensure therapy adherence [7]. Moreover, the drug’s side effects deserve attention. Glucocorticoids damage the intestinal microbiota when treatment of colitis, inhibit the immune response and intestinal barrier function, inducing sepsis [8]. Therefore, accomplishing treatment goals, reducing side effects, decreasing costs and developing new, safe and effective therapeutic strategy is a significant challenge.

The role of diets or dietary supplements in the UC prevention and long-term improvement is receiving increased attention [9, 10]. Polysaccharides isolated from purple sweet potato reduce inflammation and control intestinal flora to treat UC [11]. Similarly, *Tremella fuciformis* polysaccharides treat UC via enhancement of intestinal barrier function and anti-inflammatory effects [12]. Not only active fraction isolated from foods, but some food and nutritional supplements also exhibit beneficial effects on UC. After camel milk administration for three weeks, the intestinal inflammation in mice was significantly suppressed and intestinal flora structure restored [13]. Discovering various foods with positive effects on UC is a long-term task deserving research.

Antarctic krill (*Euphausia superba*) oil is a marine oil with healthcare functions such as anti-inflammation, neuroprotection, and cardiovascular disease risk reduction [14], which has been demonstrated beneficial for UC treatment [15–17]. However, krill oil has a complex functional composition and contains high content of phospholipid (PL) [18, 19], which poses difficulties in elucidating the therapeutic mechanism of krill oil on UC. In our previous study, the molecular species of Antarctic krill PL were characterized by UHPLC-Q-TOF–MS. A large number of polyunsaturated fatty acid groups such as EPA, DHA and AA were widely distributed in PL [20]. Among these, Antarctic krill PL (APL) can inhibit the nuclear factor kappa-B (NF-κB) pathway by regulating the activation of IκBα and phosphorylation of p65, inhibiting LPS-induced cellular inflammatory response and mouse foot swelling, which displaying great potential for UC treatment.

The fast development of gene expression profile data has made bioinformatics analysis prevalent to explore the pathogenesis of diseases. Microarray data analysis is widely used to explore differently expressed genes (DEG) and pathways crucially related to the disease, which contribute to disease diagnosis and treatment [21]. Based on bioinformatics analysis methods, the key genes, biomarkers, and action pathways of various diseases such as coronary heart disease, pulmonary embolism, and arthritis have been elucidated [22, 23]. For inflammatory bowel disease (IBD), DEG were identified between UC and Crohn's disease [24]. Certain key genes and immune cells play a vital role in the difference between the pathology of UC in adults and children [25], as well as in the progression of UC from normal to colorectal cancer [26, 27]. With the assistance of bioinformatics analysis, exploring potential therapeutic agents from functional foods towards UC and unveiling the underlying mechanisms is a promising strategy.

In this work, DEG and intersected genes of UC were found, and UC-related co-expression modules were identified through the Gene Expression Omnibus (GEO) database, Gene Cards, and Comparative Toxic Genome Database (CTD). An analysis of functional enrichment was conducted to identify crucial therapeutic targets or pathways of UC by establishing a protein–protein interaction network (PPI). On the basis of these bioinformatics analysis, the potential therapeutic effect of APL on the dextran sulfate sodium (DSS)-induced UC mouse models was thoroughly evaluated, and the underlying mechanism was uncovered through the aspects of macro disease activity index (DAI), intestinal histological damage, inflammatory cytokines, tight junction proteins, balance of intestinal flora and content of short-chain fatty acids (SCFAs). Our findings would provide an insight for the beneficial effects of APL and dietary therapy strategies for UC.

## Results

### Identification of DEG in GSE48959

In GSE48959, the normal tissue and UC samples were compared, and a total of 7060 DEG were found; of these, 3170 were significantly upregulated and 3890 were significantly downregulated. Additional file 1: Fig. S1 displays the heat map and volcano plot for these DEG.

### Weighted gene co-expression network analysis DEG from GSE48959

To identify functional clusters in UC patients, it applied WGCNA to identify stable co-expression modules associated with UC. Each module was assigned a color and represented one gene module. As shown in Additional file 1: Fig. S2A, it identified 20 modules in GSE48959. To evaluate the relationship between each module and two clinical features, a heat map of the module-feature relationship was created (Additional file 1: Fig. S2B). Each gene module comprised of several similar gene expression patterns. Additional file 1: Fig. S2C shows the blue module was the most positive correlation with UC. 3695 genes were considered to be most relevant to UC.

### Identification of intersection genes

The above data were intersected with the genes screened by CTD and Gene Cards databases, and a total of 31 intersected genes were obtained (Additional file 1: Fig. S3A). The STRING database was used to create the PPI network between overlapping genes, which excluded any genes that had no associations with other genes. The visualization produced by adding the PPI network to the Cytoscape program is shown in Additional file 1: Fig. S3B. The 10 genes with the highest scores according to the CytoHubba plug-MCC in's algorithm were EDN1 (Endothelin-1), MMP2 (72 kDa type IV collagenase), JAK2 (Tyrosine-protein kinase JAK2), ITGB2 (Integrin b2), ICAM1 (Intercellular adhesion molecule 1), TJP1 (Tight junction protein ZO-1), OCLN (Occludin), MMP9 (Matrix metalloproteinase-9), IL2RA (Interleukin-2 receptor subunit alpha), and VIM (Vimentin) (Additional file 1: Fig. S3C, D).

### Functional enrichment analyses

GO and KEGG functional enrichment analysis of these 31 genes was performed to identify potential biological functions and pathways. GO enrichment analysis includes biological processes (BPs), cellular components (CCs), and molecular functions (MFs). It showed that these genes were enriched in the regulation of cell–cell adhesion (BP), response to lipopolysaccharide (BP), external side of the plasma membrane (CC), membrane region (CC), signaling receptor activator activity (MF), and cytokine activity (MF) (Additional file 1: Fig. S4A). Meanwhile, KEGG analysis showed that these genes were enriched mainly in TNF signaling pathway, pathogenic *Escherichia coli* infection, IBD and tight junction (Additional file 1: Fig. S4B).

### APL alleviates DSS-induce colitis

**Fig. 1:**
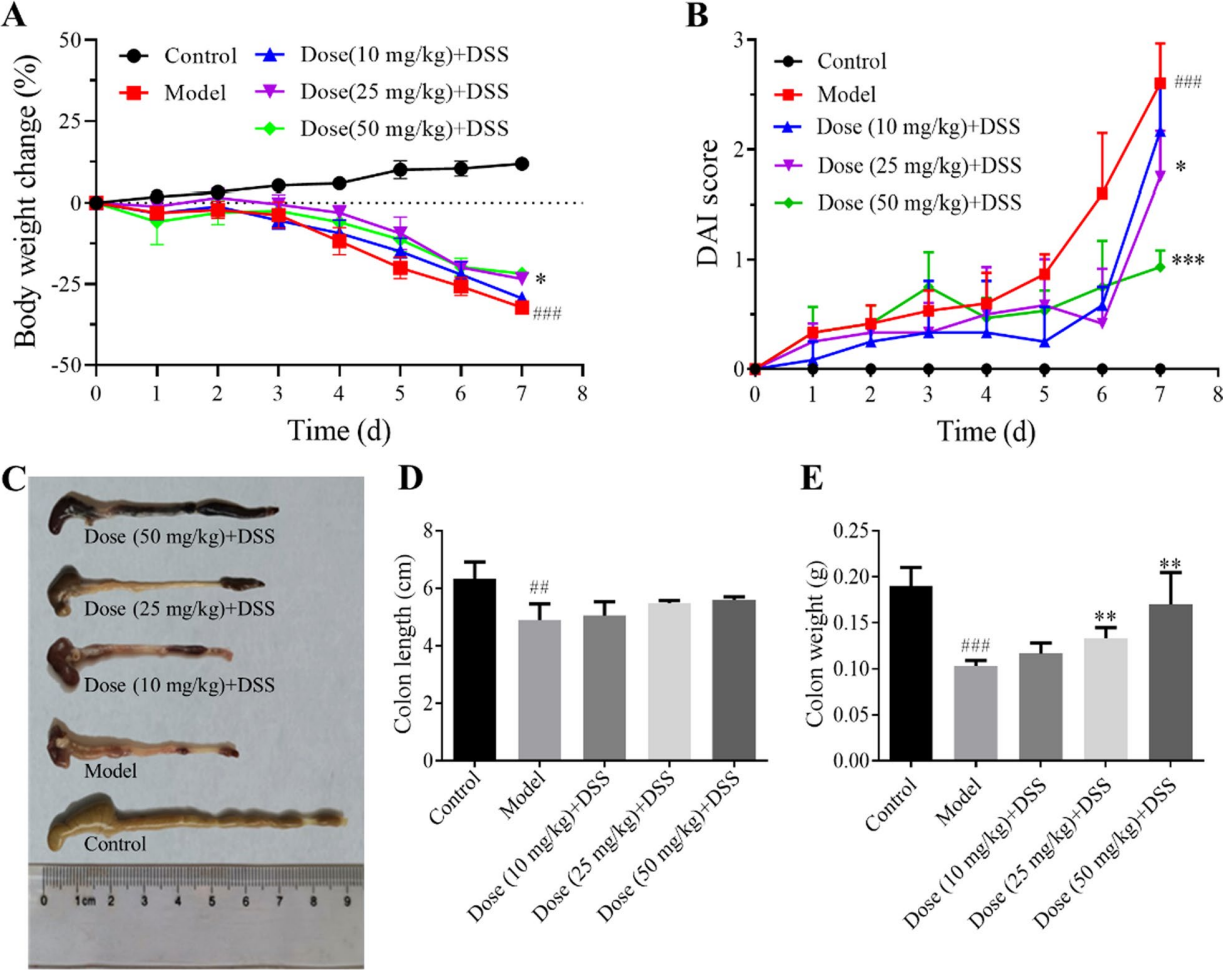
APL alleviated the symptoms of DSS-induced colitis in C57BL/6 mice. **A** Body weight of change; **B** DAI score; **C** Appearance of colon tissue; **D** Colon lengths; **E** Colon weight. All results are the mean ± SD (n = 10). ##*P* < 0.01 and ###*P* < 0.001, compared with control group; *P < 0.05, **P < 0.01 and ***P < 0.001, compared with model group

The DSS-induced colitis is a well-proven animal model that has obvious colitis symptoms such as low vitality, weight loss, decreased fecal viscosity, and stool bleeding, which were several characteristics similar to human UC patients [28, 29]. As proof of concept, a well-established DSS-induced mice colitis model was performed to evaluate the therapeutical effect of APL. As shown in Fig. 1A and B, the APL intervention treatment could effectively retard the loss of mice weight (*P* < 0.05) and reduce the DAI score (*P* < 0.001) especially in the 50 mg/kg APL group. As shown in Fig. 1C, DSS induced the atrophy of the cecum as well as the shortening, congestion, and the ulceration of colon. Moreover, APL treatment slightly improved the colon shortening (Fig. 1D) and significantly restored the colon weight (Fig. 1E) in a dose-dependent pattern (*P* < 0.01 for 25 and 50 mg/kg APL group).

### APL reduces histological inflammatory damage

**Fig. 2:**
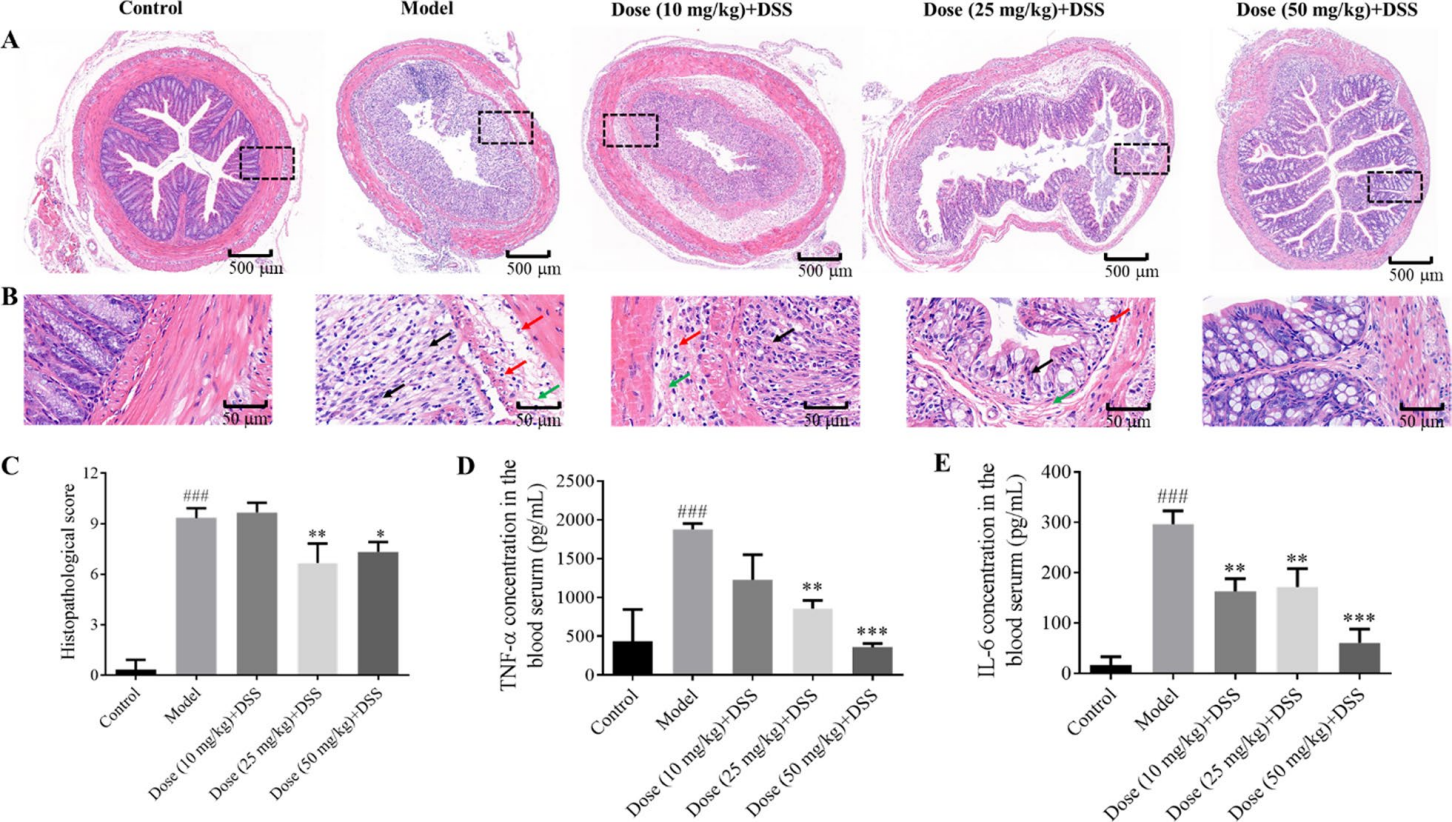
Histopathological changes of colons and inflammatory response in ulcerative colitis mice were improved by APL. **A** H&E staining images (Scale bar: 500 μm); **B** Partial enlarged details of dotted box area (black arrow: replacement of the intestinal glands with a mass of connective tissue; red arrow: inflammatory cell infiltration; green arrow: loose arrangement of muscle fibers in the muscular layer; Scale bar: 50 μm); **C** Histopathological scores; **D** Serum level of inflammatory cytokine TNF-α; **E** Serum level of inflammatory cytokine IL-6. All results are the mean ± SD (n = 3). ###*P* < 0.001, compared with control group; *P < 0.05, **P < 0.01 and ***P < 0.001, compared with model group

Figure 2A and B presented the H&E staining images of the colonic tissues upon respective treatments. The control group showed normal crypt structure, clear stratification, no injury or inflammation in the colon. By contrast in the model group, large inflammatory lesions were present in the colonic mucosa, and goblet cells were absent. As well, the inner wall of the colonic mucosa was destroyed obviously and the intestinal glands were replaced with a mass of connective tissue, accompanied by loose arrangement of muscle fibers in the muscular layer. Upon APL (10, 25, 50 mg/kg) treatment, the damage to colon tissue and infiltration of inflammatory cells were gradually reduced, and other symptoms were relieved to restore the integrity of colon structure. Histopathological scores also confirmed that APL intervention could considerably improve morphological structure of colon tissue at medium (*P* < 0.01) and high (*P* < 0.05) dosages (Fig. 2C).

### APL inhibits the pro-inflammatory cytokines expression in colonic tissue

Inflammation in the colon and excessive expression of inflammatory cytokines are caused by intestinal immune cells that are abnormally activated [30]. As shown in Fig. 2D and E, the pro-inflammatory cytokines, TNF-α and IL-6, in the serum of the model group were significantly up-regulated when compared to the control group. Whereas, the overexpression levels of TNF-α and IL-6 were efficiently inhibited upon APL intervention, indicating the strong anti-inflammatory activity of APL for potential therapy against UC.

### APL regulates the expression of intestinal tight junction protein

**Fig. 3:**
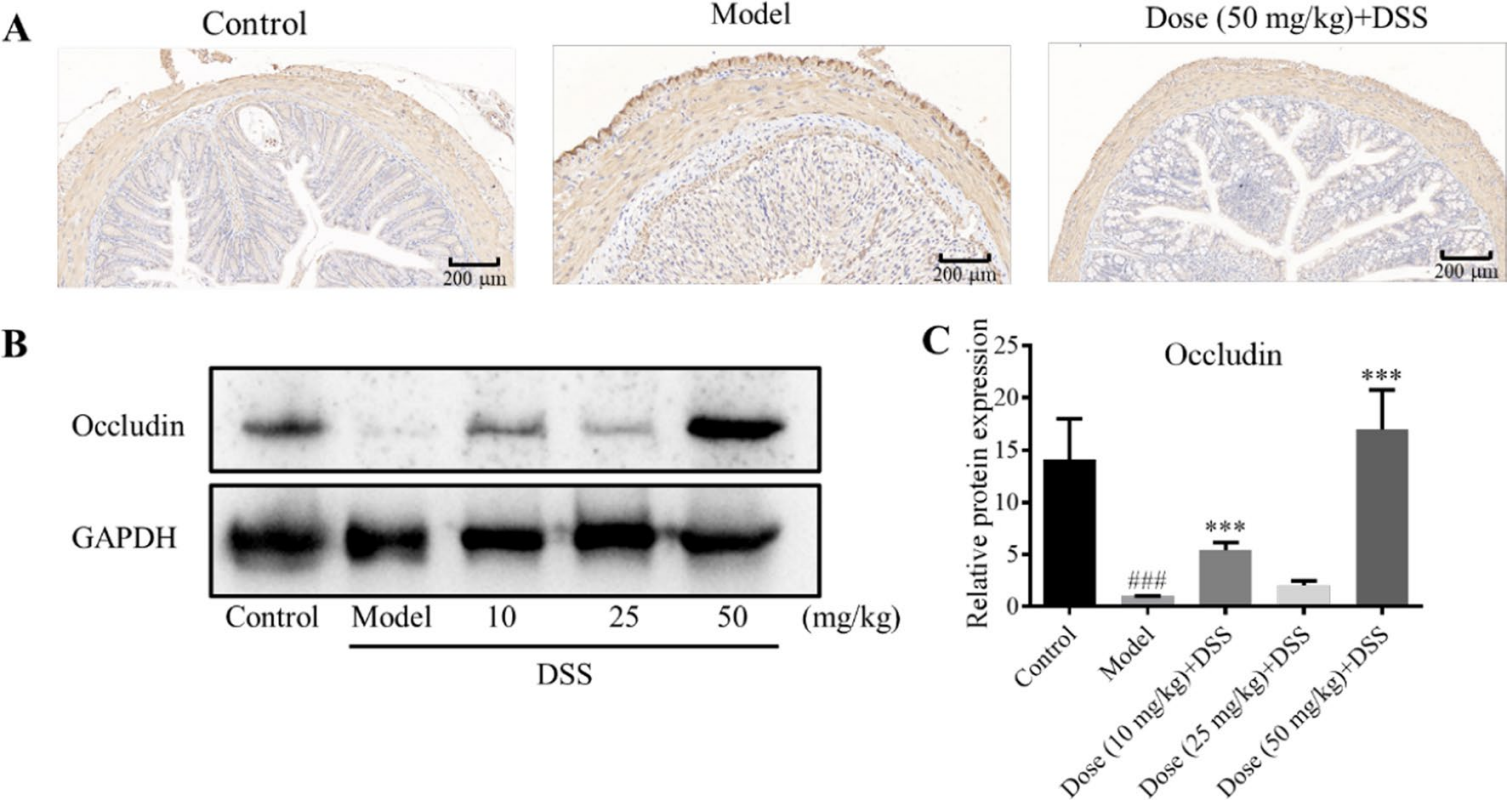
Expression of proteins involved in intestinal barrier. **A** Immunohistochemistry of DSS-induced ZO-1 secretion. The brown in the figure is the positive target. **B**, **C** Western blotting of occludin in colon tissue (**B**) and the respective gray density scanning analysis (**C**). The data shown as mean ± SD (n = 3). ###*P* < 0.001, compared with control group; ***P < 0.001, compared with model group

To evaluate the regulatory effect of APL on the intestinal epithelial barrier, the expressions of tight junction proteins, ZO-1 (Fig. 3A) and Occludin (Fig. 3B, C), were evaluated by immunocytochemistry and western-blotting, respectively [31]. It displayed that the ZO-1 expressions in the colon of the model group were significantly lower than those of the control group, while these changes were improved markedly, following the intervention with APL. Furthermore, DSS injury resulted in the decreased expression of Occludin, whereas APL treatment significantly up-regulated it.

### APL alleviates the disturbance of intestinal microbiota

**Fig. 4:**
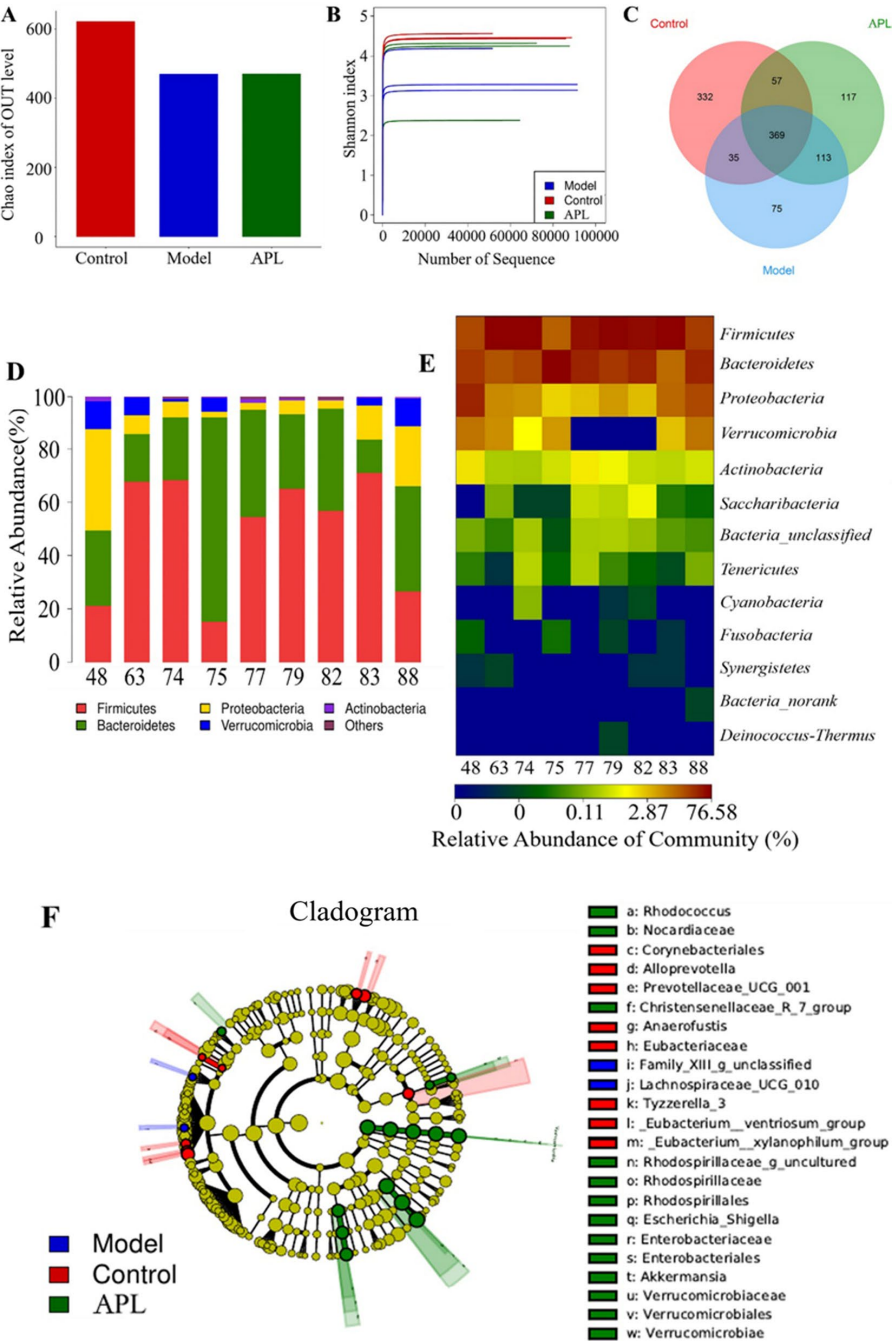
APL adjusted the composition of gut microbiota in mice. **A** Chao index; **B** Shannon index; **C** Venn diagrams; **D** Microbial community barplot; **E** Community heatmap at genus levels. **F** Cladogram plot. 77, 79, 82 were control group, 48, 83, 88 were model group, 63, 74, 75 were APL group (50 mg/kg)

The regulation of APL on the intestinal flora was investigated using 16S rDNA amplicon sequencing technology, and analyzed at the Origin-gene. By clustering the samples, the sequences are classified as OUT based on their similarity. The OTUs of the three groups were 96,946.67 ± 20,305.06, 96,238.67 ± 19,682.95, 92,544.00 ± 19,055.60. The Chao index reflects the richness of the detection samples, as shown in (Fig. 4A), the Chao index in the model group and the APL group is close to and lower than the control group. The Shannon index reflects the diversity of samples through the length of the abscissa and the ordinate and the flatness of the curve. Compared with the model group, the control group had higher species diversity and gut microbiota richness (Fig. 4B). DSS-induced UC resulted in a significant reduction in gut microbiota richness, a trend that was alleviated by APL treatment. The Venn diagram of OTUs in the three groups (Fig. 4C) showed the changes in the number of gut microbiotas. The number of species in the model group was reduced affected by UC and recovered after APL treatment. At the phylum level, the three groups *Firmicutes*, *Bacteroidetes*, *Proteobacteria*, *Verrucomicrobia*, and *Actinobacteria* contain 5 phyla with a relative abundance of intestinal flora greater than 0.01%. *Firmicutes* and *Bacteroidota* were the dominant flora. Compared with the control group, *Firmicutes* decreased and *Bacteroidota* increased in the model group. After APL treatment, *Bacteroidota* decreased and *Firmicutes* increased (Fig. 4D). The Heat map chart draws the same conclusion (Fig. 4E). LEfSe analysis revealed that the bacterial populations of the three groups differed significantly. The control group found 8 taxa, the model group found 14 taxa, and the APL group found 2 taxa (Fig. 4F).

### APL promotes the biosynthesis of SCFAs

**Fig. 5:**
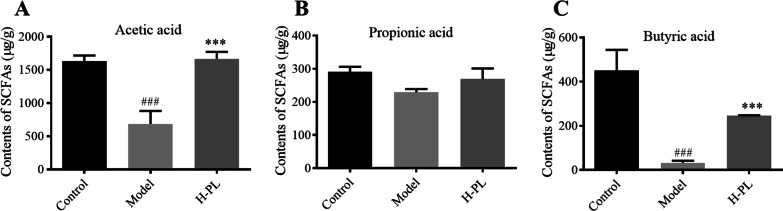
APL restored the contents of SCFAs in colon. **A** Acetic acid; **B** Propionic acid; **C** Butyric acid. The data shown as mean ± SD (n = 3). ###*P* < 0.001, compared with control group; ***P < 0.001, compared with model group

SCFAs are the main metabolites produced by the gut microbiota, which play a role in preserving colonic epithelial cells’ morphology, function, and intestinal health [32]. The control group had relatively higher levels of acetic acid, propionic acid, and butyric acid (Fig. 5), which is consistent with literature reports [33]. However, the content of SCFAs was significantly reduced after DSS induction (*P* < 0.001). Except for propionic acid, APL dramatically reversed the declining trend of acetic acid and butyric acid, which suggesting that APL could restore SCFAs to promote colon health in mice [17].

## Discussion

Although the therapeutic options for UC have evolved, among them, dietary interventions are distinctive and promising. Therefore, it is urgent to find functional food components with therapeutic effects on UC and related therapeutic mechanism investigation. This study identified 31 genes had the same expression trend in GSE48959 dataset, CTD, and Gene Crads database through comprehensive bioinformatics analysis. The top 10 genes (EDN1, MMP2, JAK2, ITGB2, ICAM1, TJP1, OCLN, MMP9, IL2RA, and VIM) associated with UC were screened according to the MCC score of the CytoHubba plugin in Cytoscape.

High concentrations of endothelin-1 (EDN1) are present in the colonic tissue of mice with Crohn's disease and UC. Local endothelin produced from inflammatory cells caused the vasculitis associated with chronic IBD via vasoconstriction and intestinal ischemia [34]. Matrix metalloproteinases (MMPs) are related to tissue injuries induced by intestinal inflammation, increased concentration of MMP-2 causing crypt hyperplasia, while MMP-9 leads to increased permeability of intestinal epithelial cell TJs exacerbating UC severity [35, 36]. Janus kinase (JAK) is critical for the transmission of cytokine types, in which JAK2 is involved in the signaling of IL-6, iNF-γ, etc. JAK inhibitors are approved by the FDA for IBD treatment currently [37]. β2-integrins (ITGB2) are required for IL-1β dependent induction of IL-22 by ILC3s, ITGB2 deficiency is associated with impaired IL-22 responses in colitis [38]. Intercellular adhesion molecule 1 (ICAM1) mediated adhesion is a prerequisite for exosome-induced T cell suppression, which expression was significantly elevated in colonic tissue of the UC mice [39, 40]. The junctional adhesion molecule and occludin are integral membrane TJ proteins, and claudins, it binds to adapters or scaffold proteins such as TJP1, TJP2, and TJP3 [41]. Reduced OCLN and ZO-1 expression, and dysbiosis of the intestinal microbiota, resulting in intestinal barrier damage [42].

GO functional enrichment analyses showed these genes were enriched in the regulation of cell–cell adhesion (BP), membrane region (CC), signaling receptor activator activity, and cytokine activity (MF). Meanwhile, KEGG revealed the TNF signaling pathway, Pathogenic Escherichia coli infection, IBD, and TJ associated with these genes. Correspondingly, UC treatment strategies currently primarily focus on the protecting of the intestinal barrier, regulating inflammatory cytokines, intestinal flora, and the immune system [6].

It has been demonstrated that the DSS-induced UC animals had elevated DAI scores, intestinal tissue disruption, and pro-inflammatory factor levels. The mechanical barrier maintains a delicate balance between keeping intestinal germs and poisons from entering and allowing for the absorption of vital nutrients, including intestinal epithelial cells, mucus layer, TJs, and lamina propria [43]. The transmembrane proteins ZO-1 and occludin are critical structures that form the TJs [44]. ZO-1 and occludin expression in colon tissue dramatically enhanced following APL treatment, indicating that APL can restore the integrity of the colonic mucosa. Similarily, In the Th2-driven porcine colitis model, KO dramatically increased the amount of goblet cells, colonic smooth muscle thickness, and overall histopathological scores [16].

More bacteria can pass across the barrier as a result of TJ disruption, which also activates macrophages and antigen-presenting cells [45]. Immune response components involved in this procedure include IgG antibodies, NF-kB, toll-like receptors, pro-inflammatory cytokines, etc. IL-1, IL-6, TNF-, T helper (Th) 1-, Th2-, and Th17-associated cytokines are expressed at comparatively higher levels in the intestinal tissues of UC patients [46]. APL treatment inhibited the overexpression of TNF-α and IL-6 and recovered their levels to normal. Phosphatidylcholine is high in KO and is attached to the mucin in colonic mucus, which forms a barrier that inhibits potentially harmful antigens from adhering to and penetrating it. Omega-3 fatty acids, however, have been demonstrated to lessen colonic inflammatory and damage by inhibiting the activation of proinflammatory transcription factors [47].

The outer mucous layer is loosely structured providing space for intestinal microorganisms to survive, while the inner layer resists colonic bacterial invasion dominated by *Bacteroidetes*, *Firmicutes*, *Epsilonbacteraeota*, and *Proteobacteria* [48]. There is an intrinsic link between UC and the gut microbiome, *Proteobacteria* and Gram-negative bacteria play a vital role in the pathogenesis of UC. Meanwhile, microbial metabolites are associated with UC progression [49]. Compared with the control group, in DSS-induced UC mice, the relative abundance of *Firmicutes* decreased, while the relative abundance of *Bacteroidetes* increased. The APL group restored the *Firmicutes/Bacteroidetes* ratio to close to the control group. This is consistent with previous studies that reported the imbalance of intestinal flora in colitis [50–52]. providing space for intestinal microorganisms to survive, while the inner layer resists colonic bacterial invasion dominated by *Bacteroidetes*, *Firmicutes*, *Epsilonbacteraeota* and *Proteobacteria* [48].

*Bacteroidetes* and *Firmicutes* phylum produce propionate, *Eubacterium rectale* and *Eubacterium hallii*, are the majority of butyrate producers. Propionate and butyrate delay the progress of inflammation and cancer of the colon [53, 54]. They primarily affect epithelial barrier function by stabilizing substances that cause hypoxia. By modulating TJs and causing the genes for claudin and occludin to be induced, butyrate also has an impact on the function of the epithelial barrier [55]. By controlling the intestinal microbiota and exerting a protective impact on the intestinal lining, APL therapy may considerably raise the intestinal SCFA contents in DSS-induced animals.

## Conclusions

In summary, DEG between UC and healthy samples were identified, among which 31 intersected genes were found to be most related to biological processes, pathways, and other critical information of UC. With this information, it investigated accordingly the positive effects and mechanisms of APL on UC. APL treatment provides significant alleviation of DSS-induced colitis by restoring the structure and function of the intestinal barrier, modulating the inflammatory response, and rebuilding the metabolites’ and guts flora's structural integrity. The results indicate APL may be a potential natural protective agent for UC treatment.

## Experimental section

### Chemicals

APL was extracted and purified from Antarctic krill oil, and its molecular species and composition were in agreement with previous reports [20]. Dextran sulfate sodium salt (DSS, MW: 40,000) was provided by Aladdin Biochemical Technology (Shanghai, China). Bicinchoninic acid (BCA) protein concentration assay kit was purchased from Beyonte Biotechnology (Shanghai, China). GAPDH Rabbit mAb, Occludin Rabbit pAb, and HRP Goat Anti-Rabbit IgG (H + L) were provided by ABclonal Technology (Wuhan, China). The ELISA kits of interleukin (IL)-6 and tumor necrosis factor (TNF)-α were supplied by Xinbosheng Biotechnology (Shenzhen, China). Urine fecal occult blood test kit were purchased from Nanjing Jiancheng Institute of Biological Engineering (Nanjing, China).

### Animals

Male C57BL/6 mice (18–22 g) were acquired from Liaoning Changsheng biological Company (Liaoning, China). It maintained all mice were maintained under standard specific pathogen-free conditions with a 12 h light/dark cycle for 1 week. The Animal Experimental Ethics Committee of South-Central MinZu University confirmed animal use and care (SYXK (Wuhan) 2016-0089, No. 2019-SCUEC-AEC-014), and all procedures performed in studies involving animals were in accordance with the ethical standards of the institution or practice at which the studies were conducted.

### Data collection

The GSE48959 microarray gene expression data were downloaded from the GEO database (http://www.ncbi.nlm.nih.gov/geo) with 8 samples of normal and 13 samples of UC. The R package “limma” was used to identify the digenome between UC and normal tissues in GSE48959 [56]. False discovery rate F < 0.05 and │log FC│ ≥ 1 were used as cut-off criteria.

Ulcerative colitis targets were obtained through the GeneCards (https://www.genecards.org) and CTD databases (https://ctdbase.org) with the keyword “ulcerative colitis”. To perform the screening, CTD: score ≥ 55; GeneCards score ≥ 7.5.

### Identifying a co-expression module by WGCNA

WGCNA is a technique for examining the expression patterns of several samples of genes. It can group genes with comparable expression patterns and examine the connection between modules and particular features or phenotypes. “WGCNA” package in R software was used to perform WGCNA [57]. The first step was to pre-process the data and evaluate its quality before creating a Pearson's correlation matrix for each pair of genes. The parameter β emphasized a strong correlation between genes and penalized weak correlations. In order to improve the similarity matrix and create a scale-free co-expression network, the appropriate β values were selected. The adjacency matrix was then transformed into a topological overlap matrix. For each gene module, the principal component analysis was performed, with module eigengenes being regarded as the largest component. Finally, the genes in the module highly related it selected to UC for subsequent analysis.

### Building and analyzing a network of protein–protein interactions (PPI)

A PPI network was created using STRING (https://string-db.org/), which searches a database of interacting genes [58]. In order to collect the PPI data, the probable targets gene list was uploaded into the STRING database with the species "Homo sapiens" and a confidence score greater than 0.7. The PPI network was then visualized using the Cytoscape 3.6.0 program [59]. The CytoHubba plug-in for Cytoscape was used to determine the MCC of each node. The 10 genes with the highest MCC scores were regarded as hub genes in this study.

### Analysis of GO and KEGG enrichment

The “clusterProfiler” package [60] has enriched Gene Ontology (GO) and Kyoto Encyclopedia of Genes and Genomes (KEGG) features. Enriched GO terms and KEGG pathways a threshold of P-value ≤ 0.05 were defined as significantly enriched terms and pathways compared to the genome-wide background.

### Therapeutic effect of APL for DSS-induced UC

#### Animal experimental design

Male c57BL/6 mice were divided randomly into five groups (n = 10) for further experiments: control group, model group (DSS) and APL groups (dose: 10, 25, 50 mg/kg) [20]. The mice in model and APL groups were treated with 3% (w/v) DSS dissolved in drinking water for 7 days, and then the mice in APL group were intragastric administered different dosages of APL. During the whole experiment, the body weight of the mice was measured daily, and the fecal status was recorded, the fecal occult blood of the mice, the length and net weight of the colon of the mice was determined.

#### Disease activity index (DAI)

It was assessed daily using the DAI based on a scoring system to assess the severity of UC, presented as mean scores for weight change, stool consistency and occult blood (Additional file 1: Table S1) [61]. The occult blood was examined using a fecal occult blood test kit.

#### Histological staining

Mice were euthanized, the colon was excised and washed with PBS solution. The length of the colon was measured and photographed. For histological analysis, the colon specimens were cut into 1 cm, and fixed in 4% paraformaldehyde and embedded in paraffin, sectioned. Then, stained with hematoxylin and eosin (H&E). Colonic tissue was analyzed using a microscope and photographed for preservation. The histopathological score was determined following a previously published method [62].

#### Immunohistochemistry

Immunohistochemistry analyzed the colonic expression of tight junction (TJ) protein ZO-1. In short, the anti-ZO-1 monoclonal antibody was used to specifically bind to the target protein in the processed tissue section, detected with biotin-labeled secondary antibody, and the sections were viewed under a microscope.

#### Determination of cytokines TNF-α, IL-6

Blood samples were collected and centrifugated to separate the serums after normal clotting. The production of TNF-α and IL-6 were measured by enzyme-linked immunosorbent assay according to the manufacturer’s protocol.

#### Western blot analysis

Approximately 100 mg of colonic tissue was lysed in ice-cold RIPA lysis buffer. The protein extraction and western blot analysis were referenced to previous reports [17].

### Gut microbiota flora analysis

The colonic contents were collected and stored at − 80 °C. The total DNA of gut microbiota was extracted by and quantified using ultraviolet spectroscopy. For a more in-depth comparatives analysis, three samples that from the control, model, and APL (50 mg/kg) group were randomly selected. From the microbial genomic DNA, specific primers were used to amplify the V3-V4 (forward primer: 5′-ACTCCTACGGGAGGCAGCAG-3′; reverse primer: 5′-GGACTACHVGGGTWTCTAAT-3′) region of the bacterial 16S rRNA gene. The composition of the gut microbiota was then determined by dual-indexing amplifica-tion and sequencing approach on the Illumina MiSeq platform followed by USEARCH (version 7.0) bioinformatics analysis. Using the MOTHUR (version v.1.30.1) clustering program, the sequences were grouped into operational taxonomic units (OTUs) at 97% sequence identity.

### Determination of contents of short-chain fatty acids (SCFA)

50 mg cecum contents were homogenized in a grinder with 15% phosphoric acid, 100 μL of 125 μg/mL internal standard solution and 400 μL of ether for 1 min. Then the supernatant was collected after centrifugation at 12,000 rpm at 4 °C for 10 min. The content of SCFAs including acetic acid, propionic acid and n-butyric acid in feces was determined by an Agilent HP-INNOWAX column (30 m × 0.25 mm × 0.25 μm) in gas chromatography system.

### Statistical analysis

All experiments were repeated three times or more, and all data were expressed as mean plus standard deviation. One-way analysis of variance (ANOVA) test was used with SPSS 20.0 to assess the analysis of differences between groups (SPSS, Chicago, IL, USA). Differences were deemed significant if their p-value was less than 0.05.

## Supplementary Information

**Additional file 1:** **Table S1.** DAI score. **Figure S1.** The results of identification of DEG in GES48959. (A) Heatmap and (B) Volcano plot of differentially expressed genes from GSE48959 (N indicates normal tissue; T indicates colitis tissue. Red represents upregulated differentially expressed genes; green represents downregulated differentially expressed genes. Black represents no significant difference genes). **Figure S2.** Identification of modules related to clinical information in GSE48959 datasets. (A) WGCNA reveals clustering and modular screening based on gene expression patterns. The top is the gene tree diagram, and the bottom is the gene modules in different colors; (B) Clarified the overlap between the modules. Each row and each column correspond to a module. The colors in the table indicate the gene counts at the intersection of the corresponding modules; (C) The correlation between MEblue membership and gene significance. **Figure S3.** Construction of the PPI network and screening of hub genes. (A) Venn diagram used to identify crossgenes; (B) PPI network constructed by the STRING database; (C) The PPI network is visualized by Cytoscape software. The blue nodes represent the genes. Edges indicate interaction associations between nodes. (D) Identification of the hub genes from the PPI network by the maximum clique centrality (MCC) algorithm. Highlighted nodes represent genes with the highest MCC sores. **Figure S4. **31 genes were analyzed by GO and KEGG. (A) GO analysis; (B) KEGG pathway analysis. GO analysis includes biological processes (BPs), cellular components (CCs), and molecular functions (MFs). The count represents the number of genes and the color represents the adjusted p-value. *P *< 0.05.

## Data Availability

The data that support the findings of this study were available on request from the corresponding author, upon reasonable request.
