# Correction: Protective effect and mechanism insight of purified Antarctic krill phospholipids against mice ulcerative colitis combined with bioinformatics

**DOI:** 10.1007/s13659-023-00379-y

**Published:** 2023-05-06

**Authors:** Rong Huang, Jiaxu Yao, Li Zhou, Xiang Li, Jinrui Zhu, Yueqi Hu, Jikai Liu

**Affiliations:** School of Pharmaceutical Sciences, National Demonstration Center for Experimental Ethnopharmacology Education, South-Central MinZu University, Wuhan, 430074 People’s Republic of China

**Correction** **:** **Natural Products and Bioprospecting (2023) 13:11** 10.1007/s13659-023-00375-2

Following publication of the original article [1], the authors reported that “krill” was mistakenly written as “kill” in the title.

The correct title should read:

Protective effect and mechanism insight of purified Antarctic krill phospholipids against mice ulcerative colitis combined with bioinformatics

The original article [1] has been updated.
