# Supplementary material for: Protective effect and mechanism insight of purified Antarctic krill phospholipids against mice ulcerative colitis combined with bioinformatics
Source: Nat Prod Bioprospect. 2023 Apr 5;13(1):11. doi: 10.1007/s13659-023-00375-2 (PMC10073399; doi:10.1007/s13659-023-00375-2)
Supplement: Supplementary file 1 — Additional file 1: Table S1. DAI score. Figure S1. The results of identification of DEG in GES48959. (A) Heatmap and (B) Volcano plot of differentially expressed genes from GSE48959 (N indicates normal tissue; T indicates colitis tissue. Red represents upregulated differentially expressed genes; green represents downregulated differentially expressed genes. Black represents no significant difference genes). Figure S2. Identification of modules related to clinical information in GSE48959 datasets. (A) WGCNA reveals clustering and modular screening based on gene expression patterns. The top is the gene tree diagram, and the bottom is the gene modules in different colors; (B) Clarified the overlap between the modules. Each row and each column correspond to a module. The colors in the table indicate the gene counts at the intersection of the corresponding modules; (C) The correlation between MEblue membership and gene significance. Figure S3. Construction of the PPI network and screening of hub genes. (A) Venn diagram used to identify crossgenes; (B) PPI network constructed by the STRING database; (C) The PPI network is visualized by Cytoscape software. The blue nodes represent the genes. Edges indicate interaction associations between nodes. (D) Identification of the hub genes from the PPI network by the maximum clique centrality (MCC) algorithm. Highlighted nodes represent genes with the highest MCC sores. Figure S4. 31 genes were analyzed by GO and KEGG. (A) GO analysis; (B) KEGG pathway analysis. GO analysis includes biological processes (BPs), cellular components (CCs), and molecular functions (MFs). The count represents the number of genes and the color represents the adjusted p-value. P < 0.05. [file 13659_2023_375_MOESM1_ESM.docx]

**Additional Information**

**Table S1 DAI score**

| Body weight loss | Stool consistency | Blood in stool | score |
| --- | --- | --- | --- |
| none | normal | negative | 0 |
| 1-5% |  |  | 1 |
| 5-10% | loose stools | positive | 2 |
| 10-15% |  |  | 3 |
| over 15% | diarrhea | gross rectal bleeding | 4 |

**Figure Captions**

**Figure S1** The results of identification of DEG in GES48959. (A) Heatmap and (B) Volcano plot of differentially expressed genes from GSE48959 (N indicates normal tissue; T indicates colitis tissue. Red represents upregulated differentially expressed genes; green represents downregulated differentially expressed genes. Black represents no significant difference genes).

**Figure S2** Identification of modules related to clinical information in GSE48959 datasets. (A) WGCNA reveals clustering and modular screening based on gene expression patterns. The top is the gene tree diagram, and the bottom is the gene modules in different colors; (B) Clarified the overlap between the modules. Each row and each column correspond to a module. The colors in the table indicate the gene counts at the intersection of the corresponding modules; (C) The correlation between MEblue membership and gene significance.

**Figure S3** Construction of the PPI network and screening of hub genes. (A) Venn diagram used to identify cross genes; (B) PPI network constructed by the STRING database; (C) The PPI network is visualized by Cytoscape software. The blue nodes represent the genes. Edges indicate interaction associations between nodes. (D) Identification of the hub genes from the PPI network by the maximum clique centrality (MCC) algorithm. Highlighted nodes represent genes with the highest MCC sores.

**Figure S4** 31 genes were analyzed by GO and KEGG. (A) GO analysis; (B) KEGG pathway analysis. GO analysis includes biological processes (BPs), cellular components (CCs), and molecular functions (MFs). The count represents the number of genes and the color represents the adjusted p-value. P< 0.05.


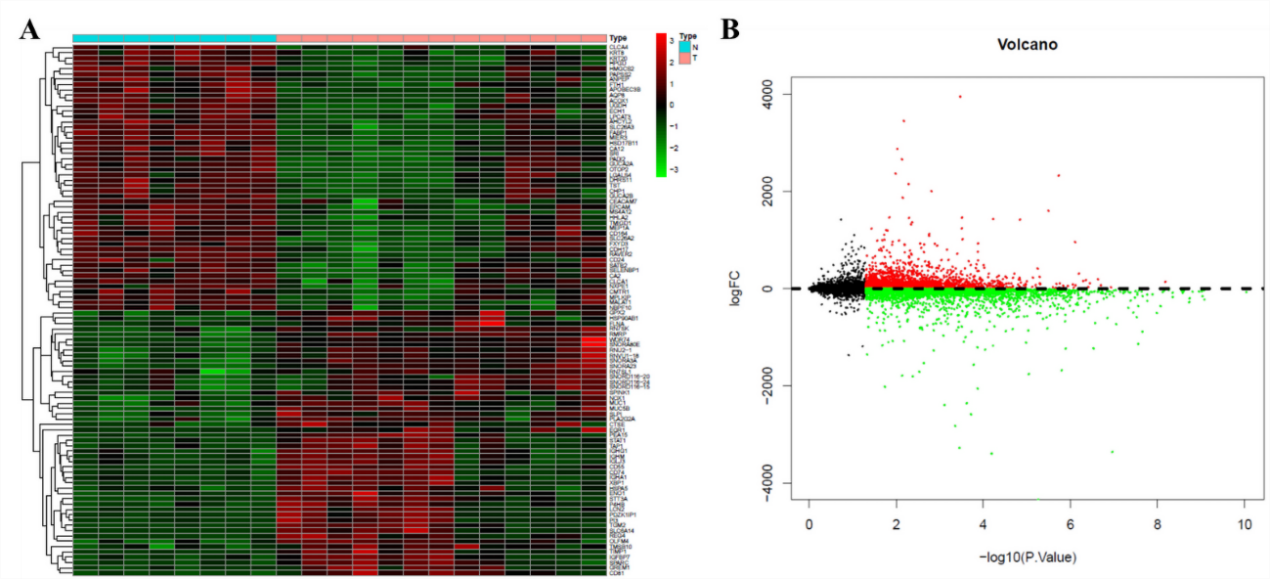


**Figure S1**


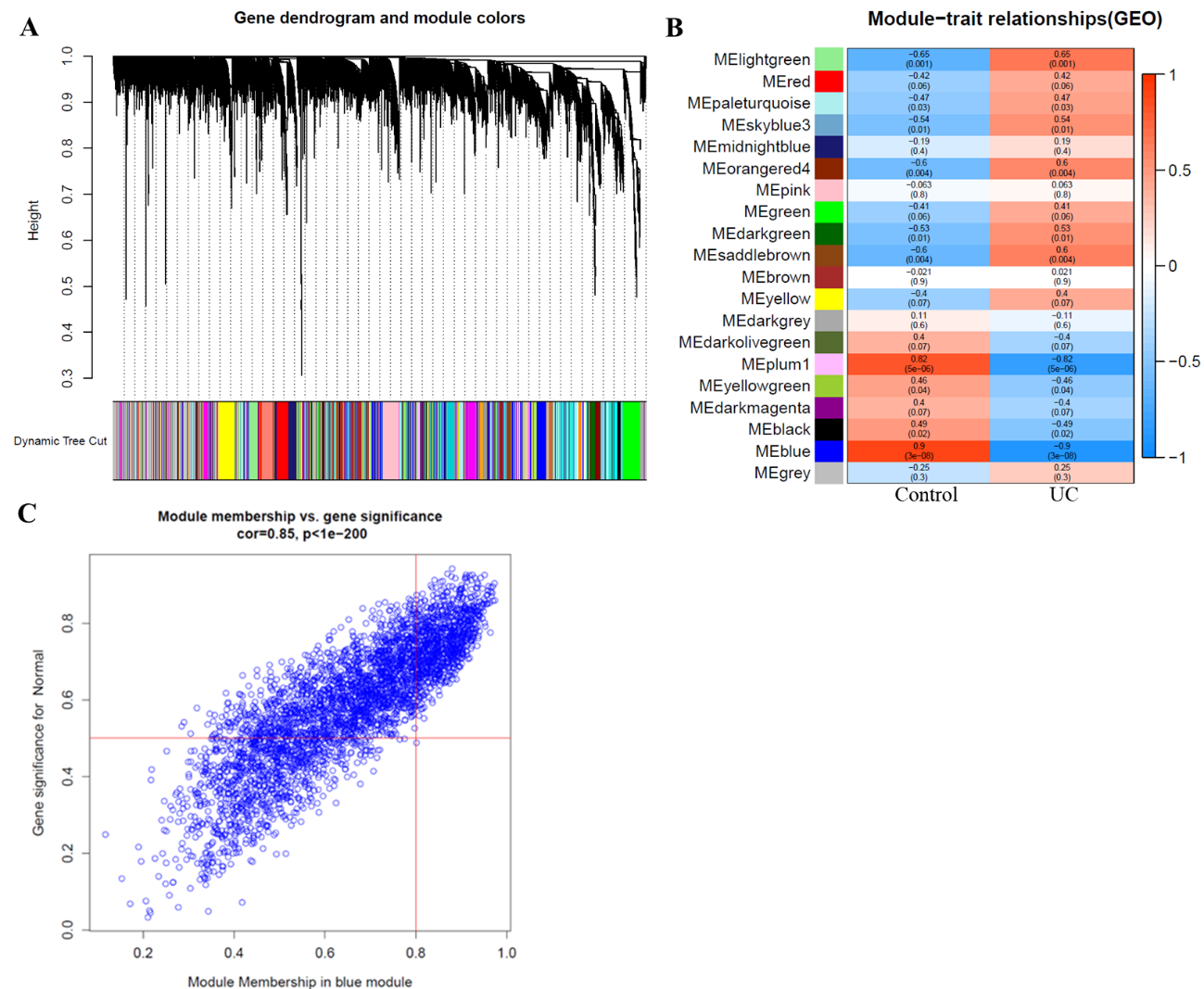


**Figure S2**


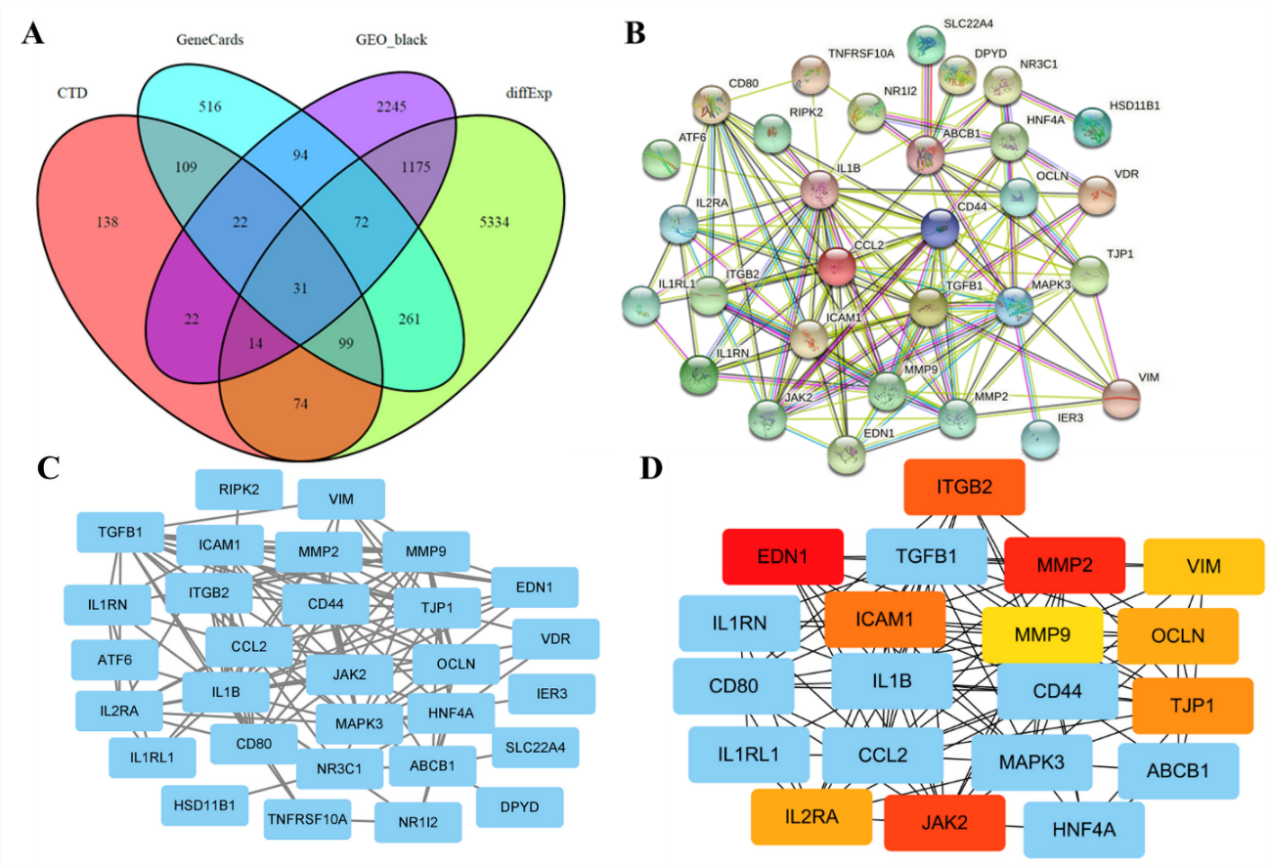


**Figure S3**


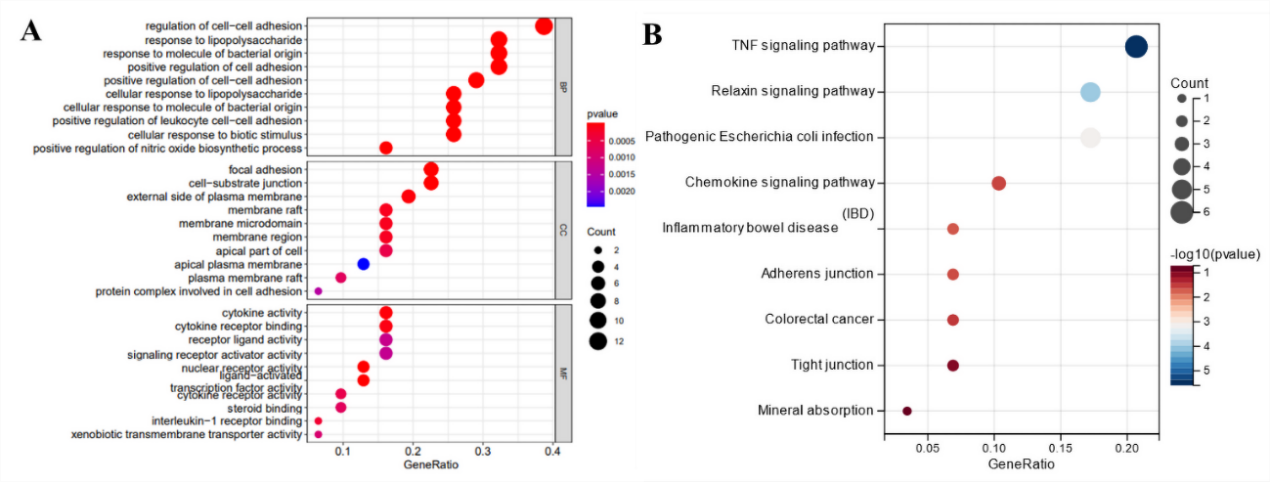


**Figure S4**
